# Functional liver imaging score derived from hepatobiliary-specific contrast-enhanced MRI: a study on agreement and correlation

**DOI:** 10.1186/s13244-025-02107-1

**Published:** 2025-11-19

**Authors:** Hou-yun Xu, Xi-ping Yu, Xiao-chao Yu, Xuan Jin, Lu-ping Wang, Ji-bo Hu, Hong-jie Hu

**Affiliations:** 1 Department of Radiology, The Fourth Affiliated Hospital of School of Medicine, and International School of Medicine, International Institutes of Medicine, Zhejiang University, Yiwu, China; 2 Department of Pathology, The Fourth Affiliated Hospital of School of Medicine, and International School of Medicine, International Institutes of Medicine, Zhejiang University, Yiwu, China; 3 Department of Radiology, Sir Run Run Shaw Hospital, Zhejiang University School of Medicine, Hangzhou, China; 4 Medical Imaging International Scientific and Technological Cooperation Base of Zhejiang Province, Zhejiang, China

**Keywords:** Functional liver imaging scores, Magnetic resonance imaging, Chronic liver disease, Liver function evaluation, Gadoxetic acid

## Abstract

**Purpose:**

To investigate inter-and intra-observer agreement of Functional Liver Imaging Score (FLIS) in different populations, hepatobiliary phase (HBP), and radiologists, while analyzing the correlation between FLIS and liver function.

**Methods:**

A single-center retrospective study analyzed 203 patients from 2017 to 2021. Inter-observer and intra-observer consistency was assessed by the Intraclass Correlation Coefficient (ICC) by means of Spearman correlation analysis, evaluating the correlation between FLIS and Child-Turcotte-Pugh (CTP) score, as well as the relevant laboratory data. The discriminatory efficacy of FLIS for different stages of CLD and CTP grades was assessed by the receiver operating characteristic curve.

**Results:**

In all 203 patients, inter-observer ICC range was 0.885 to 0.954, and intra-observer ICC range was 0.946 to 0.985 among different radiologists. Inter-observer ICC range was 0.908 to 0.985, and intra-observer ICC range was 0.924 to 0.991 at different HBP time points. Inter-observer ICC range was 0.89 to 1, and intra-observer ICC range was 0.943 to 1 in different populations. The correlation coefficients between FLIS and albumin, total bilirubin, international normalized ratio, prothrombin time, and CTP scores were 0.617, −0.651, −0.706, −0.724, and −0.818. FLIS had good diagnostic efficacy in differentiating different stages of CLD and CTP grades; the area under the curve was 0.708, 0.752, 0.871, and 0.908.

**Conclusions:**

FLIS had a good intra-observer and inter-observer agreement among different populations, HBP and radiologists. FLIS showed a good correlation with CTP grades and laboratory data. FLIS can be used as one of the imaging assessment tools to distinguish different stages of CLD and CTP grades.

**Critical relevance statement:**

FLIS showed significant correlations with prothrombin time, international normalized ratio, serum albumin, total bilirubin, and CTP score, and distinguished different stages of CLD and CTP grades, which positioned it as a non-invasive imaging tool for liver function assessment.

**Key Points:**

- High reliability: FLIS demonstrates excellent inter- and intra-observer agreement among different radiologists, hepatobiliary phase (10–25 min), and populations (healthy subjects, CLD, cirrhosis).
- Strong correlation with liver function: FLIS significantly correlates with liver function markers: albumin, total bilirubin, international normalized ratio, prothrombin time, and CTP scores.
- Diagnostic efficacy: differentiates CLD stages and CTP grades, highest accuracy for distinguishing advanced cirrhosis.
- Clinical utility: FLIS serves as a robust and non-invasive imaging tool for assessing liver function and stratifying disease severity in CLD and cirrhosis.

**Graphical Abstract:**

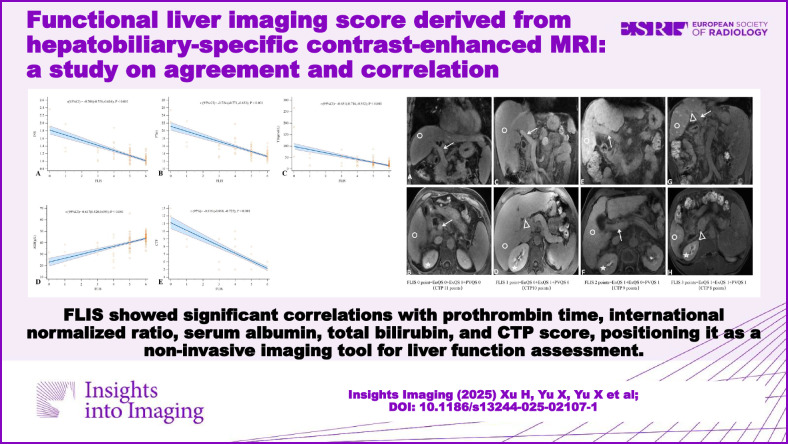

## Introduction

Assessment of liver function is critically important for personalized management of CLD and hepatic tumors, as well as for preoperative evaluation in liver transplantation [1–8]. In clinical practice, the assessment of liver disease severity and functional impairment predominantly relies on clinical signs and blood biochemical markers, such as alanine aminotransferase (ALT), aspartate aminotransferase (AST), serum albumin (ALB), total bilirubin (TB), prothrombin time (PT), along with manifestations like ascites and hepatic encephalopathy [9, 10]. Furthermore, several standardized liver function scoring systems are widely employed, including the Albumin-Bilirubin (ALBI) score, CTP, Model for End-Stage Liver Disease (MELD) score, and the Indocyanine Green Retention Test at 15 min (ICG-R15) [11–17].

Although the aforementioned laboratory parameters and liver function grading systems are instrumental in guiding clinical decisions, they have notable limitations. The ALBI inadequately reflects the true functional status of end-stage liver disease. Furthermore, its reliance on albumin and bilirubin levels may be confounded by nutritional status or biliary obstruction, leading to inaccuracies [11]. The CTP contains five equally weighted parameters: TB, ALB, PT, ascites, and hepatic encephalopathy. However, these parameters are susceptible to extraneous influences, and the grading of ascites and hepatic encephalopathy involves subjective evaluation [11]. The MELD incorporates TB, creatinine, and the International Normalized Ratio (INR), but fails to account for complications related to portal hypertension. Moreover, non-hepatic factors may alter TB, INR, or creatinine levels, compromising its validity [13]. Despite being regarded as the “gold standard” for assessing functional hepatic reserve, the ICG-R15 test requires prolonged testing time, lacks real-time liver imaging information, and is prone to interference from hepatic blood flow or biliary excretion, limiting its accuracy [16].

In recent years, Bastati et al [18–20] developed FLIS, a semi-quantitative MRI-based scoring system for hepatic function evaluation. FLIS quantifies liver function by summing three sub-scores derived from gadoxetic acid-enhanced MRI HBP features: the liver parenchyma Enhancement Quality Score (EnQS), the biliary contrast Excretion Quality Score (ExQS), and the Portal Vein Signal Quality Score (PVQS). Previous studies [21, 22] have demonstrated strong correlations between FLIS and conventional scoring systems (CTP, ALBI). However, critical gaps remain in the current literature: the reproducibility of FLIS across different HBP and diverse patient populations has not been systematically investigated. Furthermore, its broader applicability for liver function assessment remains unvalidated. This study aims to address these gaps through two primary objectives: ① to evaluate the agreement of FLIS in different HBP time points and patient populations; ② to analyze the correlation between FLIS and laboratory-based liver function indices, exploring its discriminatory capacity for CLD stratification and CTP classification.

## Methods

### Research subject

**Fig. 1:**
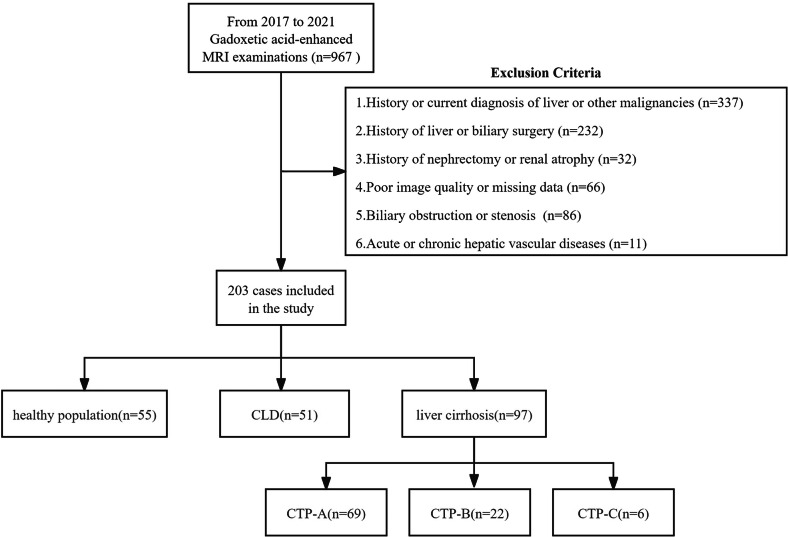
Flow diagram of study population

Using the Electronic Medical Record System (EMRS) and picture archiving and communication system, we retrospectively analyzed 967 patients who underwent gadoxetic acid-enhanced MRI at our hospital from June 2017 to December 2021. Inclusion criteria [19, 23]: ① HBP images at 10, 15, 20, and 25 min; ②Patients who did not receive therapeutic anticoagulation; ③Diagnosis of CLD or cirrhosis based on histopathology, imaging, or clinical evidence. Diagnostic criteria of CLD : (1) Clinical diagnostic criteria: presence of persistent liver injury factors for ≥ 6 months, including but not limited to: viral hepatitis, alcohol abuse, metabolic associated fatty liver disease, autoimmune hepatitis, schistosomiasis liver disease; (2) Imaging diagnostic criteria: Evidence of morphological alterations on ultrasound (US), computed tomography (CT), or magnetic resonance imaging (MRI), such as: left lobe enlargement/right lobe atrophy, heterogeneous hepatic parenchymal echogenicity/density/signal intensity, absence of definitive cirrhotic nodules or unequivocal signs of portal hypertension (e.g., splenomegaly, portosystemic collateral vessels). Compensated cirrhosis diagnostic criteria: meeting one of the following criteria: liver biopsy demonstrating pseudolobule formation and fibrous septa partitioning the hepatic parenchyma; evidence of esophageal or gastric varices or portosystemic collateral vessel formation via endoscopy; US, CT, or MRI presenting characteristic features: irregular/nodular liver surface contour, hepatic lobe disproportion (e.g., caudate lobe hypertrophy), widened hepatic fissures, splenomegaly, portal vein trunk diameter ≥ 13 mm. For cases lacking definitive evidence, cirrhosis could be diagnosed if two of the following five criteria were met [24–27]: (1) platelet count (PLT) < 100 × 10^9^/L without other explanations; (2) ALB < 35 g/L, excluding malnutrition or renal diseases; (3) INR > 1.3; (4) aspartate aminotransferase-to-platelet ratio index (APRI) > 2; (5) Fibrosis 4 Index (FIB-4) > 1.3. Diagnostic criteria for decompensated cirrhosis: established cirrhosis accompanied by complications of portal hypertension and impaired hepatic function: ascites, esophageal or gastric variceal hemorrhage, hepatic encephalopathy, and so on. Exclusion criteria [28–30]: ① prior or current malignancies in the liver or other organs (*n* = 337); ② history of hepatobiliary surgery (*n* = 232); ③ nephrectomy or renal atrophy (*n* = 32); ④ poor image quality (*n* = 21); ⑤ biliary obstruction or stenosis (*n* = 86); ⑥ acute or chronic hepatic vascular diseases (*n* = 11); ⑦ incomplete laboratory data within 2 weeks of MRI (*n* = 45). Ultimately, 203 subjects were enrolled (Fig. 1).

The study was conducted retrospectively in accordance with the Declaration of Helsinki (as revised in 2013). Written informed consent was obtained from all participants prior to MRI, and the study was approved by the Committee on Human Subject Research and Ethics of The Fourth Affiliated Hospital of Zhejiang University School of Medicine (Ethics approval number: K2024206; date of approval: January 18, 2024).

### Clinical data

The demographic information and laboratory data were obtained from the EMRS. The clinical information consisted of the age, gender, weight, height, and epidemiological causes of liver disease; laboratory tests included PLT, estimated glomerular filtration rate, serum creatinine, INR, AST, ALT, alkaline phosphatase (ALP), gamma-glutamyl transferase (GGT), TB, PT, ALB, and CTP score. Based on the CTP score, liver function is divided into three grades: CTP -A (5–6 points): mild liver function impairment; CTP -B (7–9 points): moderate liver function impairment; CTP -C (10–15 points): severe liver function impairment.

### MRI examination protocol

All gadoxetic acid-enhanced MRIs were performed using a 1.5-T scanner (GE, Signa HDxt), equipped with a 16-channel phased-array body coil. Detailed scanning parameters are listed in Supplementary Table 1. The imaging protocol included the following sequences, Non-enhanced sequences: T1-weighted imaging (in-phase and out-of-phase), 2D magnetic resonance cholangiopancreatography, Pre-contrast fat-suppressed T1WI, Fat-suppressed T2-weighted imaging, Diffusion-weighted imaging (DWI, b-values = 0, 200, and 800 s/mm^2^); Enhanced dynamic scans: arterial phase (18–20 s), portal venous phase (60–70 s), and delayed phase (80–90 s). HBP imaging: axial images at 10, 15, 20, and 25 min, additional coronal plane imaging at 20-min HBP. Contrast agent: Gadoxetic acid disodium (Primovist, Bayer, Germany, 10 mL). Injection protocol: A dose of 10 mL (0.025 mmol/kg) was administered intravenously at a rate of 1.0 mL/s, followed immediately by 20 mL saline flush.

### Image analysis

**Fig. 2:**
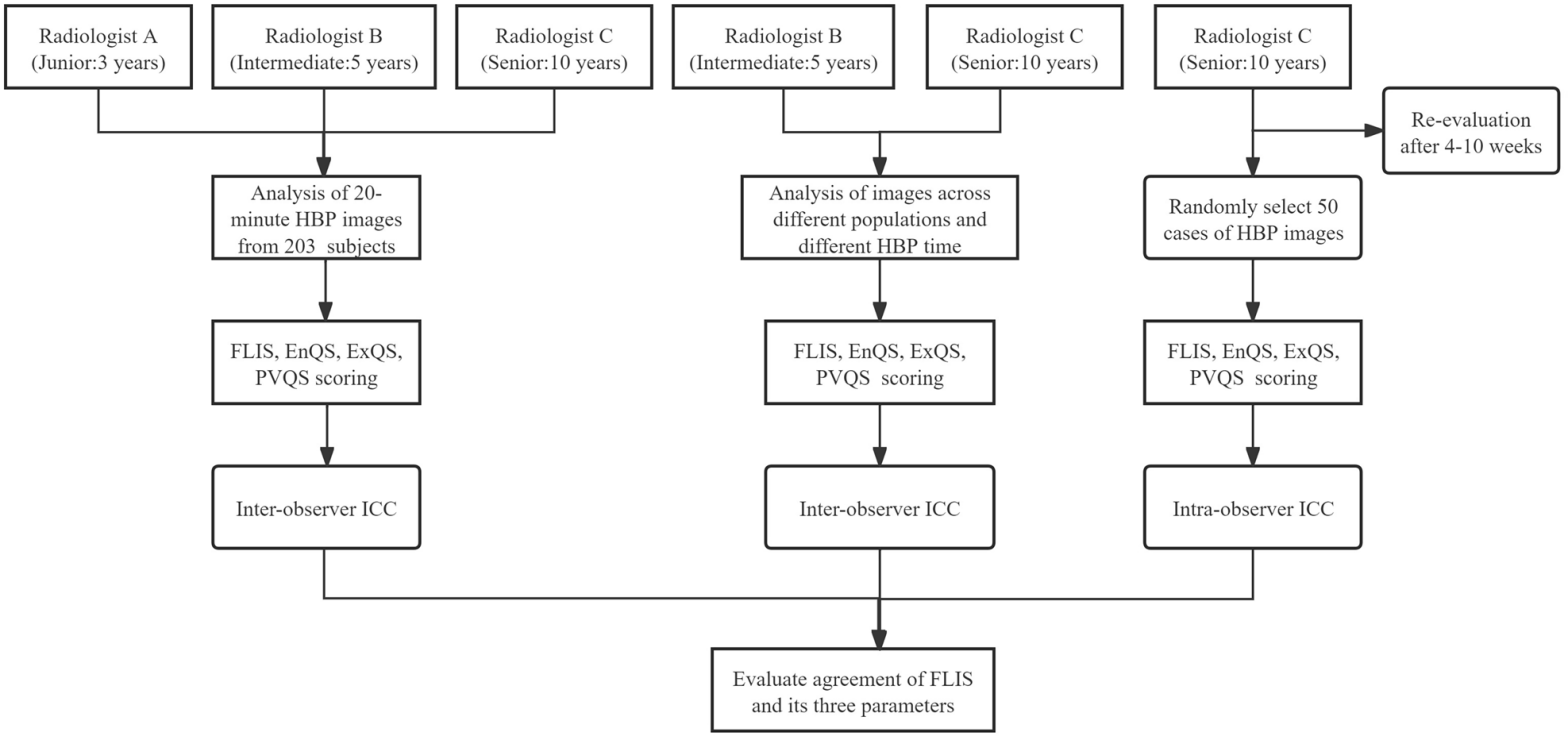
Flowchart of image analysis

**Fig. 3:**
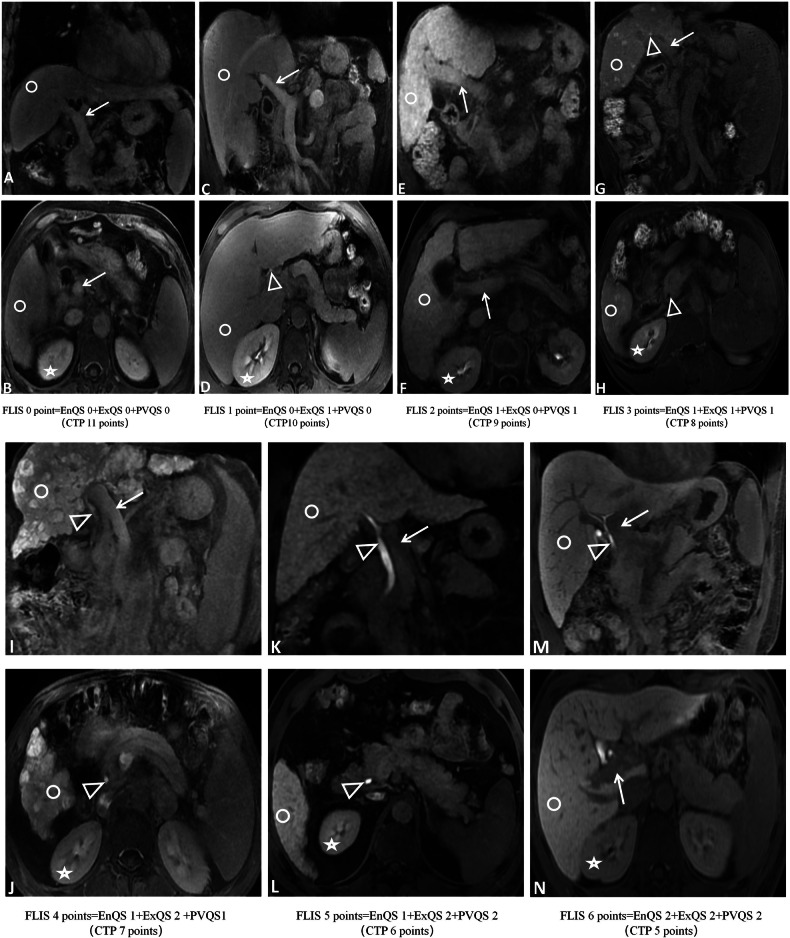
Comparison of FLIS and CTP scores in 20-min HBP images of 7 patients with CLD and cirrhosis. **A**, **B** Male, 58 years old, alcoholic decompensated cirrhosis, CTP score of 11 points (CTP -C). In the 20-min HBP, the right kidney signal and portal vein signal were higher than the hepatic parenchymal signal (both EnQS and PVQS scored 0 points). No contrast excretion was observed in intra- or extrahepatic bile ducts (ExQS 0 point). FLIS : 0 points. **C**, **D** Male, 48 years old, alcoholic decompensated cirrhosis, CTP score of 10 points (CTP -C). In the 20-min HBP, the right kidney signal and portal vein signal were higher than the hepatic parenchymal signal (both EnQS and PVQS scored 0 points). Contrast excretion was observed in intrahepatic bile ducts (ExQS 1 point). FLIS: 1 point. **E**, **F** Male, 72 years old, alcoholic decompensated cirrhosis, CTP score of 9 points (CTP -B). In the 20-min HBP, the right kidney signal and portal vein signal were equal to the hepatic parenchymal signal (both EnQS and PVQS scored 1 point). No contrast excretion was observed in intra- or extrahepatic bile ducts (ExQS 0 point). FLIS: 2 points. **G**, **H** Male, 63 years old, hepatitis B-related decompensated cirrhosis, CTP score of 8 points (CTP -B). In the 20-min HBP, the right kidney signal and portal vein signal were equal to the hepatic parenchymal signal (both EnQS and PVQS scored 1 point). Contrast excretion was observed in intrahepatic bile ducts (ExQS 1 point). FLIS: 3 points. **I**, **J** Male, 59 years old, hepatitis B-related decompensated cirrhosis, CTP score of 7 points (CTP -B). In the 20-min HBP, the right kidney signal and portal vein signal were equal to the hepatic parenchymal signal (both EnQS and PVQS scored 1 point). Contrast excretion was observed in the common bile duct (ExQS 2 points). FLIS: 4 points. **K**, **L** Male, 45 years old, hepatitis B-related compensated cirrhosis, CTP score of 6 points (CTP -A). In the 20-min HBP, the right kidney signal was equal to the hepatic parenchymal signal (EnQS 1 point), while the portal vein signal was lower than the hepatic parenchymal signal (PVQS 2 points). Contrast excretion was observed in the common bile duct (ExQS 2 points). FLIS: 5 points. **M**, **N** Male, 32 years old, hepatitis B-related compensated cirrhosis, CTP score of 5 points (CTP -A). In the 20-min HBP, the right kidney signal and portal vein signal were lower than the hepatic parenchymal signal (both EnQS and PVQS scored 2 points). Contrast excretion was observed in the common bile duct (ExQS 2 points). FLIS: 6 points

All HBP images from 203 subjects were independently and blindly evaluated by three radiologists with varying diagnostic experience levels (Radiologist A: junior, 3 years; Radiologist B: intermediate, 5 years; Radiologist C: senior, 10 years) using PACS system (Fig. 2). Prior to analysis, the three radiologists underwent standardized training, including independent evaluation of HBP images from 50 cases to align interpretation criteria. Subsequently, they independently assessed all images. Inter-observer agreement for FLIS and its three subparameters across different time points and subgroups was analyzed by Radiologists B and C. To assess intra-observer agreement, Radiologist C re-evaluated images and randomly selected a subset of 50 patients’ images 4–10 weeks after the initial assessment [31, 32]. Representative FLIS images in cirrhotic patients are illustrated in Fig. 3, and a detailed description of FLIS is shown in Supplementary Table 2. FLIS, evaluated by the more experienced Radiologist C, was utilized for statistical analysis.

### Statistical analysis

Data were analyzed using SPSS (version 27.0, IBM). Normality of data distribution was tested using the Kolmogorov-Smirnov test. Normally distributed continuous variables were expressed as mean ± standard deviation, and between-group differences were analyzed using ANOVA. Non-normally distributed continuous variables and ordinal categorical data were expressed as median and interquartile range (IQR), with between-group differences assessed using the Kruskal–Wallis test. Categorical variables were described as counts and percentages, and analyzed with the chi-square test. Inter-and intra-observer agreement was evaluated using ICC: ICC ≤ 0.4, poor agreement; 0.4 < ICC ≤ 0.6, moderate agreement; 0.6 < ICC ≤ 0.8, good agreement; ICC > 0.8, excellent agreement.

Spearman correlation analysis was used to assess relationships between FLIS and three subgroups, CTP, as well as laboratory parameters. Receiver operating characteristic (ROC) curve analysis was performed to evaluate the diagnostic performance of FLIS in distinguishing CTP grades, healthy individuals from CLD, and cirrhotic from non-cirrhotic cases. The Youden index was used to determine optimal cutoff thresholds. Diagnostic accuracy was assessed by the area under the curve (AUC), sensitivity, and specificity. To account for multiple testing across the performed statistical tests (including several ROC analyses), we applied a Bonferroni correction. This adjusted the significance level from *p* = 0.05 to *p* = 0.0125 (0.05 divided by four).

## Results

A total of 203 subjects were included in the retrospective analysis, comprising 133 males (65.52%) and 70 females (34.48%). The mean age was 49.51 ± 11.79 years (range: 23–81 years), and the mean body mass index (BMI) was 23.24 ± 2.73 kg/m². Among the 203 subjects, 55 (27.09%) were categorized into the healthy control group, 51 (25.13%) into the CLD group, and 97 (47.78%) into the liver cirrhosis group. The cirrhotic cohort included 69 cases CTP -A, 22 cases CTP -B, and 6 cases CTP -C. Comprehensive baseline clinical characteristics of the study population are presented in Supplementary Table 3.

### Inter-and intra-observer agreement analysis of FLIS and its three subparameters in different radiologists

The inter- and intra-observer ICC results for FLIS and three subparameters among all 203 subjects were summarized in Table 1. The inter-observer ICC among radiologists A, B, and C all exceeded 0.85, indicating excellent agreement. Compared to the three subparameters, FLIS demonstrated the highest inter-observer consistency, with an ICC of 0.954 (95% confidence interval [CI]: 0.942–0.964). For intra-observer agreement, the ICC values for FLIS and three subparameters were all above 0.9. Notably, FLIS achieved the highest intra-observer consistency, with an ICC of 0.985 (95% CI: 0.980–0.988).

**Table 1:** Intra- and inter-observer agreement for FLIS and its three subparameters in different radiologists

Parameters	Inter-observer agreement			Intra-observer agreement		
	ICC	(95% CI)	*p*-value	ICC	(95% CI)	*p*-value
EnQS	0.896	(0.869, 0.919)	< 0.001	0.946	(0.928, 0.959)	< 0.001
ExQS	0.908	(0.884, 0.928)	< 0.001	0.981	(0.975, 0.986)	< 0.001
PVQS	0.885	(0.854, 0.910)	< 0.001	0.971	(0.962, 0.978)	< 0.001
FLIS	0.954	(0.942, 0.964)	< 0.001	0.985	(0.980, 0.988)	< 0.001

*EnQS* enhancement quality score, *ExQS* excretion quality score, *PVQS* portal vein signal quality score, *FLIS* functional liver imaging score, *ICC* intraclass correlation coefficient

### Inter-and intra-observer agreement analysis of FLIS and its three subparameters at different HBP

The intra- and inter-observer ICC analyses for FLIS and its three subparameters across multiple HBP time points (10, 15, 20, and 25 min) in all 203 subjects are shown in Tables 2 and 3. All inter-observer ICC values exceeded 0.9, indicating excellent agreement. Specifically, inter-observer ICCs at 20-min and 25-min HBP were slightly higher than those at 10-min and 15-min HBP. FLIS consistently demonstrated higher inter-observer ICC values than its individual subparameters across all timepoints, achieving its maximum value of 0.985 (95% CI: 0.980–0.989) at 20-min HBP. Similarly, all intra-observer ICC values exceeded 0.9 at different HBP timepoints, indicating excellent consistency. Mirroring the inter-observer ICC, FLIS showed higher intra-observer ICC values than its subparameters at all timepoints, with the highest ICC value of 0.991 (95% CI: 0.988–0.993), recorded at 20-min HBP.

**Table 2:** Inter-observer agreement for FLIS and its three subparameters at different HBP

Parameters	ICC (95% CI)				*p*-value
	10 min	15 min	20 min	25 min	
EnQS	0.916 (0.891, 0.936)	0.931 (0.910, 0.947)	0.954 (0.940, 0.965)	0.975 (0.968, 0.981)	< 0.001
ExQS	0.908 (0.877, 0.930)	0.946 (0.929, 0.959)	0.953 (0.938, 0.964)	0.944 (0.926, 0.957)	< 0.001
PVQS	0.922 (0.897, 0.941)	0.938 (0.919, 0.953)	0.972 (0.964, 0.979)	0.969 (0.959, 0.976)	< 0.001
FLIS	0.925 (0.902, 0.942)	0.957 (0.943, 0.967)	0.985 (0.980, 0.989)	0.982 (0.976, 0.986)	< 0.001

*HBP* hepatobiliary phase, *EnQS* enhancement quality score, *ExQS* excretion quality score, *PVQS* portal vein signal quality score, *FLIS* functional liver imaging score, *ICC* intraclass correlation coefficient

**Table 3:** Intra-observer agreement for FLIS and its three subparameters at different HBP

Parameters	ICC (95% CI)				*p*-value
	10 min	15 min	20 min	25 min	
EnQS	0.951 (0.937, 0.963)	0.969 (0.960, 0.977)	0.977 (0.970, 0.983)	0.986 (0.982, 0.990)	< 0.001
ExQS	0.924 (0.901, 0.942)	0.968 (0.958, 0.976)	0.967 (0.957, 0.975)	0.962 (0.950, 0.971)	< 0.001
PVQS	0.937 (0.917, 0.953)	0.951 (0.935, 0.962)	0.981 (0.976, 0.986)	0.979 (0.973, 0.984)	< 0.001
FLIS	0.955 (0.941, 0.966)	0.975 (0.968, 0.981)	0.991 (0.988, 0.993)	0.990 (0.986, 0.992)	< 0.001

*HBP* hepatobiliary phase, *EnQS* enhancement quality score, *ExQS* excretion quality score, *PVQS* portal vein signal quality score, *FLIS* functional liver imaging score, *ICC* intraclass correlation coefficient

### Inter-and intra-observer agreement analysis of FLIS and its three subparameters in different populations

The 203 subjects were categorized into three groups: healthy population, CLD, and cirrhosis. Inter- and intra-observer ICC analyses for FLIS and its three subparameters across these groups were detailed in Table 4.

**Table 4:** Inter-and intra-observer agreement of FLIS and its three subparameters in different populations

Parameters	Inter-observer ICC (95% CI)				Intra-observer ICC (95% CI)			
	HI	CLD	Cirrhosis	*p*-value	HI	CLD	Cirrhosis	*p*-value
EnQS	1	0.890 (0.824, 0.933)	0.903 (0.864, 0.932)	< 0.001	1	0.952 (0.915, 0.972)	0.956 (0.934, 0.971)	< 0.001
ExQS	1	0.908 (0.854, 0.945)	0.922 (0.891, 0.946)	< 0.001	1	1	0.943 (0.916, 0.962)	< 0.001
PVQS	1	0.896 (0.834, 0.937)	0.902 (0.863, 0.931)	< 0.001	1	1	0.967 (0.950, 0.978)	< 0.001
FLIS	1	0.910 (0.857, 0.946)	0.956 (0.938, 0.969)	< 0.001	1	0.952 (0.915, 0.972)	0.980 (0.970, 0.987)	< 0.001

*HI* healthy individuals, *CLD* chronic liver disease; *EnQS* enhancement quality score, *ExQS* excretion quality score, *PVQS* portal vein signal quality score, *FLIS* functional liver imaging score

Inter-observer agreement: healthy population group, both FLIS and its subparameters demonstrated perfect agreement (ICC = 1.0); In the CLD group, all subparameters and FLIS exceeded 0.85, with FLIS showing the highest ICC (0.910; 95% CI: 0.857–0.946); In the Cirrhosis group, all subparameters and FLIS exceeded 0.9, with FLIS again demonstrating the highest ICC (ICC = 0.956; 95% CI: 0.938–0.969).

Intra-observer agreement: healthy population group, both FLIS and all subparameters demonstrated perfect agreement (ICC = 1.0). In the CLD group, the intra-observer ICC values of ExQS and PVQS were 1, while FLIS and EnQS were 0.95. In the cirrhosis group, all subparameters and FLIS demonstrated excellent agreement (ICC > 0.9), with FLIS achieving the highest ICC (0.980; 95% CI: 0.970–0.987).

### Analysis of differences in clinical characteristics, imaging parameters, and laboratory data

Supplementary Table 4 presents the differences in clinical baseline characteristics, laboratory data, FLIS and its three subparameters across the three groups. The results indicate that, in addition to creatinine, eGFR, and ALT, significant statistical differences (all *p* < 0.05) were observed among the three groups in terms of age, gender, BMI, TB, ALB, INR, PT, AST, ALP, GGT, and PLT. These findings suggest that, after accounting for similar renal function levels (excluding the influence of abnormal renal function), there were notable differences in liver function-related laboratory data among the three groups. Furthermore, FLIS and EnQS showed statistically significant differences among the three groups (*p* < 0.001), whereas ExQS and PVQS did not.

### Spearman correlation analysis of FLIS and its three subparameters with different populations, CTP, and laboratory data

**Fig. 4:**
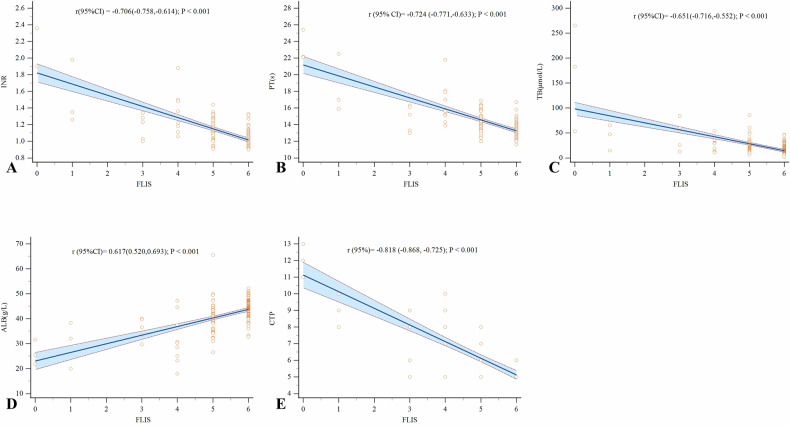
Scatter plots of Spearman correlation analysis between FLIS and partial laboratory parameters/CTP score. FLIS showed strong negative correlations with INR (r = −0.706, **A**), PT (r = −0.724, **B**), TB (r = −0.651, **C**), and CTP score (r = −0.818, **E**), along with a positive correlation with ALB (r = 0.617, **D**), all statistically significant (*p* < 0.001). These results demonstrate that FLIS exhibits strong correlations with INR, PT, TB, ALB, and CTP score

The Spearman correlation analysis showed that FLIS, EnQS, ExQS, and PVQS exhibited certain correlations with different populations, with FLIS and EnQS demonstrating the strongest correlations, with correlation coefficients r of −0.525 and −0.532. In contrast, the correlation coefficients r for ExQS and PVQS were both below −0.3. Analysis of the correlation between CTP score and FLIS revealed that FLIS and its three subparameters all had strong correlations with CTP score, with correlation coefficients r of −0.676, −0.617, −0.808, and −0.818. Among these, FLIS showed the strongest correlations. Analysis of the correlation between FLIS and its three subparameters with laboratory data showed that TB, ALB, INR, PT, AST, ALP, GGT, and PLT all exhibited certain correlations. Among these, PT had the highest correlation coefficient, with r values of −0.724, suggesting that FLIS can reflect liver function to some extent. Supplementary Table 5 presents the above analysis results. Figure 4 displays the Spearman correlation analysis scatter plots of FLIS with INR, PT, TB, ALB, and CTP scores.

### Diagnostic efficacy of FLIS in CLD Stratification: a subparameter analysis

The efficacy analysis of FLIS and its three subparameters in distinguishing between healthy populations and CLD showed that EnQS and FLIS exhibited AUC values of 0.704 and 0.708, these differences were statistically significant (*p* < 0.001). In contrast, ExQS and PVQS demonstrated no statistically significant efficacy in differentiating these two groups. For the differentiation between cirrhotic and non-cirrhotic populations, EnQS and FLIS showed moderate discriminative value, with statistically significant AUC values of 0.745 and 0.752 (*p* < 0.001).

**Fig. 5:**
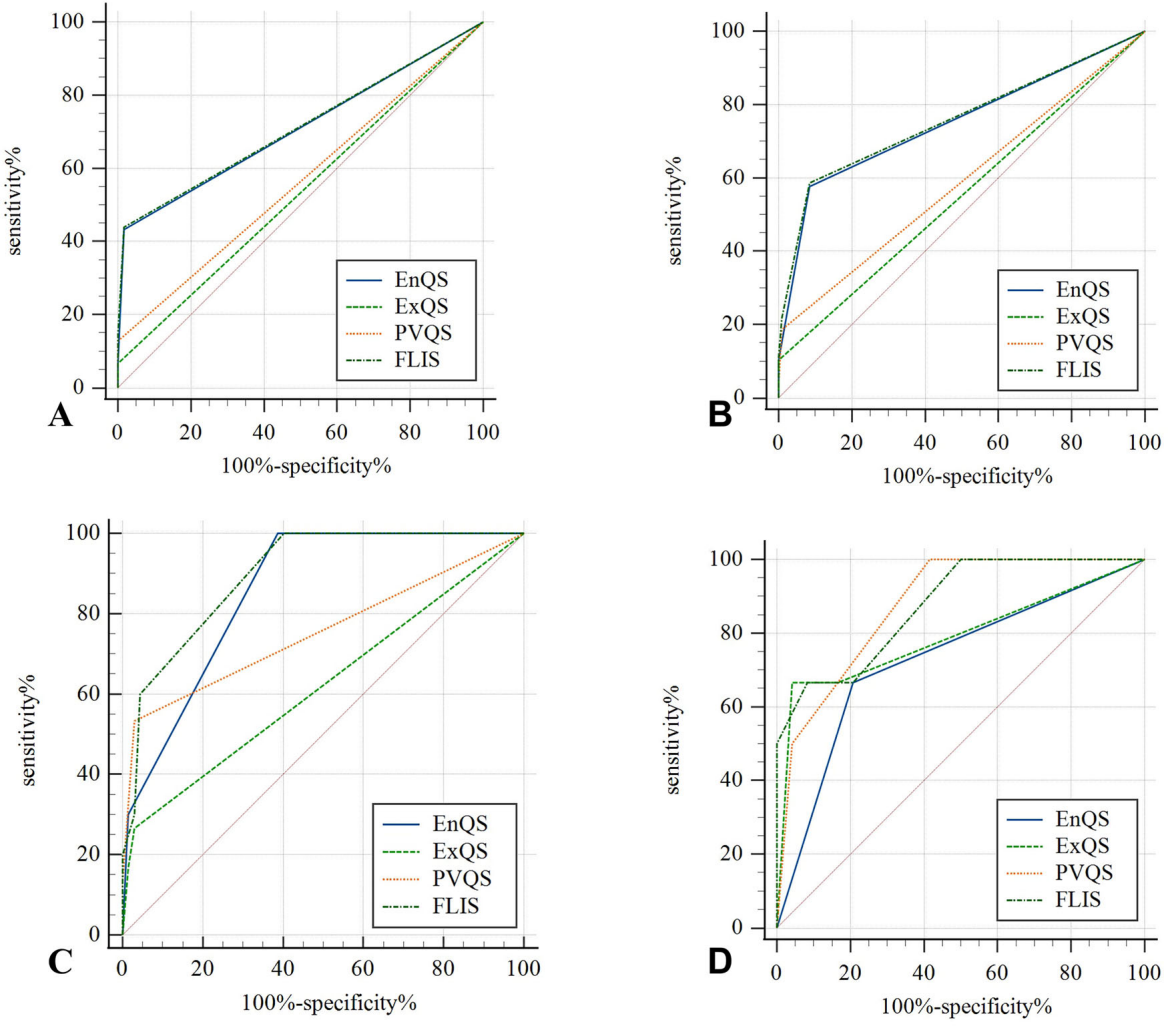
ROC curve of EnQS, ExQS, PVQS, and FLIS in discriminating different populations and CTP classifications. **A** The performance in discriminating between healthy individuals and chronic liver disease; **B** the efficacy in distinguishing cirrhotic patients from non-cirrhotic patients; **C** the ability to differentiate CTP -A from CTP - (**B**, **C**); **D** the discriminative capacity between CTP -B and CTP -C

In the analysis of CTP classification, EnQS, PVQS, and FLIS demonstrated high clinical discriminative value for distinguishing CTP -A from CTP -(B and C), with AUC values of 0.857, 0.773, and 0.908, respectively (*p* < 0.001). However, ExQS showed the AUC value of 0.619, with no statistical significance (*p* = 0.067). For distinguishing CTP -B from CTP -C, the AUC values of FLIS and its three subparameters were 0.729, 0.795, 0.864, and 0.871, respectively. Notably, PVQS and FLIS showed statistically significant discriminative efficacy, while EnQS and ExQS did not. These findings (summarized in Table 5 and Fig. 5) indicate that FLIS and its three subparameters exhibit varying diagnostic performance in differentiating populations and CTP classifications, with FLIS demonstrating the highest overall efficacy across all categories.

**Table 5:** Efficacy analysis of FLIS and its three subparameters in differentiating between healthy individuals and chronic liver disease, cirrhotic and non-cirrhotic, and CTP classification

Groups	AUC	95% CI	S.E	Cutoff	Sensitivity%	Specificity%	*p*-value
Differentiation between HI and CLD							
EnQS	0.704	(0.634, 0.775)	0.036	1.5	98.2	42.6	**< 0.001**
ExQS	0.527	(0.44, 0.614)	0.045	1.5	100	5.4	0.554
PVQS	0.564	(0.48, 0.648)	0.043	1.5	100	12.8	0.16
FLIS	0.708	(0.638, 0.778)	0.036	5.5	98.2	43.2	**< 0.001**
Differentiation between cirrhotic and non-cirrhotic							
EnQS	0.745	(0.676, 0.815)	0.036	1.5	91.5	56.7	**< 0.001**
ExQS	0.541	(0.462, 0.621)	0.041	1.5	100	8.2	0.311
PVQS	0.588*	(0.509, 0.667)	0.04	1.5	99.1	18.6	0.03
FLIS	0.752	(0.683, 0.821)	0.035	5.5	91.5	57.7	**< 0.001**
Differentiation between CTP -A and CTP -(B and C)							
EnQS	0.857	(0.789, 0.931)	0.036	1.5	60.9	100	**< 0.001**
ExQS	0.619	(0.486, 0.752)	0.068	1.5	98.6	25	0.067
PVQS	0.773	(0.653, 0.893)	0.061	1.5	97.1	57.1	**< 0.001**
FLIS	0.908^†^	(0.849, 0.966)	0.03	5.5	59.4	100	**< 0.001**
Differentiation between CTP -B and CTP -C							
EnQS	0.729	(0.474, 0.965)	0.125	0.5	77.3	66.7	0.104
ExQS	0.795	(0.548, 1)	0.126	0.5	95.5	66.7	0.029
PVQS	0.864	(0.716, 1)	0.075	1.5	54.5	100	**0.007**
FLIS	0.871	(0.712, 1)	0.081	2	90.9	66.7	**0.006**

After applying a correction for multiple testing (Bonferroni method: 0.05/4), statistical significance was defined as *p* < 0.0125

*CTP* Child-Turcotte-Pugh classification, *EnQS* enhancement quality score, *ExQS* excretion quality score, *PVQS* portal vein quality score, *FLIS* functional liver imaging score, *HI* healthy individual

* Lowest diagnostic efficacy

^†^ Highest diagnostic efficacy

The bold values indicate statistically significant differences (*p* < 0.01)

## Discussion

Although some current studies [22, 30] have demonstrated the clinical value of FLIS in liver function evaluation and prediction of post-hepatectomy liver failure, there remains a need to address critical clinical questions regarding its applicability across diverse populations, its correlation with laboratory liver function, and the consistency of assessment across various demographic groups. This study conducted a single-center retrospective analysis of HBP imaging data from 203 subjects, yielding the following findings: ① FLIS and its three subparameters demonstrated excellent consistency across different populations, HBP, and radiologists with varying levels of experience, supporting its broad clinical application in diverse populations including healthy individuals, CLD, and cirrhotic; ② FLIS showed significant correlations with laboratory liver function markers and CTP score, particularly demonstrating correlation coefficients of 0.724 with PT and 0.818 with CTP score, indicating its potential as a reliable non-invasive imaging tool for liver function assessment; ③ FLIS demonstrated strong discriminative ability in differentiating CLD stages and CTP classifications, the AUC value of 0.908 in distinguishing CTP -A from CTP -(B and C). These findings suggest that FLIS represents a valuable stratification tool for hepatic function grading in cirrhotic patients, providing critical decision-making support for clinicians in formulating stage-specific management strategies for CLD.

This study comprehensively demonstrated the consistency and stability of FLIS through multidimensional analysis of ICC. The consistency of ICC among radiologists with different experience levels revealed that both inter-observer (all exceeding 0.85) and intra-observer ICC (all exceeding 0.9) for FLIS and its three subparameters remained high. Notably, even for the less-experienced radiologist, the lowest inter-observer ICC reached 0.885, indicating that FLIS maintains robust stability even when assessed by junior radiologists. These findings further confirmed that FLIS demonstrated broad applicability independent of radiologists’ experience, facilitating its adoption in primary healthcare institutions. These results align with the work of Bastati et al [19], who reported inter-observer and intra-observer ICC of 0.93 and 0.98 for FLIS, respectively, closely mirroring our observed results of 0.954 and 0.985.

In the consistency analysis across different HBP, both intra- and inter-observer ICC were above 0.9 at all time intervals. Remarkably, even at the 10-min HBP, the inter-group ICC reached 0.908, with ICC progressively increasing over time. However, minimal ICC differences emerged between HBP 20-min and 25-min. Notably, extending to HBP-25 min provided limited additional benefit for ICC improvement. These findings suggested that clinical protocols could optimize MRI efficiency by reducing HBP duration (e.g., to 10 or 15 min) for liver function evaluation without compromising consistency.

The population-based ICC analysis demonstrated exceptional consistency across cohorts: perfect ICC (1.0) in healthy populations, ICC exceeding 0.85 in CLD, and ICC surpassing 0.9 in cirrhotic patients. This robust cross-population agreement highlights FLIS as a promising tool for liver function assessment across disease stages, warranting future research to systematically validate its diagnostic accuracy in stage-stratified CLD management.

This analysis of FLIS and liver function-associated laboratory parameters revealed significant correlations with multiple biochemical indices, particularly demonstrating strong associations with PT and INR (r > 0.7), and moderate correlations with TB and ALB (r > 0.5), which indicates FLIS can reflect hepatic coagulation and synthetic functions. Importantly, the 203 subjects had no comorbidities affecting PT, INR, TB, or ALB, confirming FLIS validity in assessing hepatic reserve capacity. However, weaker correlations emerged between FLIS and AST, ALT, ALP, GGT, and PLT. This likely stems from multiple confounding factors: fluctuations in transaminase levels across different phases of hepatitis B progression, variations during quiescent hepatic fibrosis versus active cirrhosis; discrepancies in laboratory instrumentation affecting measurement standardization; and inherent variability in platelet enumeration. These inherent limitations reduce the reliability of AST, ALT, ALP, GGT, and PLT as precise markers of real-time hepatic function [33, 34], explaining their diminished correlation with FLIS.

FLIS also had clinical application value in distinguishing stages of CLD. This study found that FLIS demonstrated diagnostic efficacy with an AUC of 0.708 in differentiating healthy individuals from CLD. The optimal cutoff threshold was 5.5 points (falling between 4 and 5 points), suggesting some overlap in FLIS, which necessitates further validation with larger-scale studies to confirm its utility in early diagnosis of CLD. In distinguishing cirrhosis from non-cirrhotic conditions, although FLIS showed higher discriminative performance with an AUC of 0.752, the clinical discriminative value of FLIS remains modest for early CLD stages. Whether it holds potential as an imaging monitoring tool for tracking progression from CLD to cirrhosis warrants further research.

Additionally, FLIS and its three subparameters exhibited strong correlations with CTP scores, with the highest correlation coefficient r −0.818, consistent with the −0.8 reported by Lee et al [22] This supported FLIS as a functional stratification biomarker for cirrhotic. Notably, FLIS demonstrated excellent discrimination between CTP classifications: an AUC of 0.908 for differentiating CTP - A from CTP - (B and C), and an AUC of 0.871 for distinguishing between CTP -B and CTP -C. Although the results lack external validation and calibration analysis and the results for CTP-B vs C are underpowered (only 6 CTP-C cases), we believe this is still a good preliminary exploratory study and provides direction for future research. Since most compensated cirrhotic patients belong to CTP -A, while decompensated cases typically belong to CTP -B or C, accurate CTP classification using FLIS could enhance early management for cirrhotic patients, provide warnings for decompensation events.

This study has several important limitations. First, it is a single-center retrospective study, which inherently carries the risk of selection bias, and the exclusion of patients with hepatic malignancies may also introduce selection bias and compromise the generalizability of our findings to this specific population. While efforts were made to mitigate this—such as including healthy individuals, CLD patients, and cirrhotic patients, and performing multidimensional consistency assessments (radiologist experience levels, different HBP times, and varying stages of CLD)—these measures cannot fully eliminate bias. Second, the number of CTP-C patients included was very small (*n* = 6), resulting in an unbalanced distribution across CTP classes. This limited the robustness of subgroup comparisons and the reliability of diagnostic performance estimates in more advanced disease stages. The authors acknowledge this and propose to expand this subgroup in future work; however, the current results for CTP-B vs CTP-C must be interpreted with caution. Thirdly, a portion of the CLD and cirrhosis diagnoses were not pathologically confirmed but were based on clinical, laboratory, and imaging criteria. While this approach reflects common clinical practice, it introduces potential diagnostic uncertainty, particularly in earlier disease stages. Additionally, the use of laboratory values obtained up to 2 weeks apart from imaging may introduce temporal variability in correlations with liver function scores. Finally, the study employed a single 1.5-T MRI scanner model, which may limit the generalizability of the results to other field strengths or vendors.

While our study focused on serum biomarkers and FLIS in liver function assessment, we acknowledge the emerging role of quantitative MRI in liver assessment. Techniques like T1 mapping and elastography offer promising avenues for non-invasive quantification of hepatic fibrosis and inflammation. T1 relaxometry can be useful in diagnosing patients with impaired liver function or chronic liver disease, which have a good relationship with the Child–Pugh classification, MELD and ICG clearance test. MRE-based liver stiffness measurement emerges as a robust prognostic tool in chronic liver disease management and a non-invasive technique for liver fibrosis evaluation. Additionally, quantitative T1 reduction rate has emerged as a complementary tool for liver function assessment. As Obmann et al [35] demonstrated, the T1 reduction rate provides field-strength-independent quantification that strongly correlates with CTP staging and MELD scores. This approach offers absolute quantification of hepatocellular function, effectively complementing semi-quantitative systems like FLIS within the expanding toolkit of MRI-based functional liver evaluation.

## Conclusion

The findings of this study demonstrated that FLIS exhibited strong consistency and stability across radiologists with varying levels of experience, different HBP times, and various stages of CLD. FLIS showed favorable correlations with laboratory indicators related to hepatic coagulation and synthetic function. It enabled stratified classification for patients with CLD and cirrhosis, aiding clinicians in developing management and follow-up strategies. FLIS held potential as a novel non-invasive imaging method for the assessment of liver function.

## Supplementary information

ELECTRONIC SUPPLEMENTARY MATERIAL

## Data Availability

The study’s data can be obtained from the corresponding author upon reasonable request. Data are provided within the manuscript.

## Abbreviations

ALB: Albumin
ALBI: Albumin-bilirubin
ALP: Alkaline phosphatase
ALT: Alanine aminotransferase
AST: Aspartate transaminase
CLD: Chronic liver disease
CTP: Child-Turcotte-Pugh
EnQS: Enhancement quality score
ExQS: Excretion quality score
FLIS: Functional liver imaging score
HBP: Hepatobiliary phase
HI: Healthy individuals
ICC: Intraclass correlation coefficient
INR: International normalized ratio
MRI: Magnetic resonance imaging
PT: Prothrombin time
PVQS: Portal vein signal quality score
TB: Total bilirubin
