# Supplementary material for: Functional liver imaging score derived from hepatobiliary-specific contrast-enhanced MRI: a study on agreement and correlation
Source: Insights Imaging. 2025 Nov 19;16:259. doi: 10.1186/s13244-025-02107-1 (PMC12630446; doi:10.1186/s13244-025-02107-1)
Supplement: Supplementary file 1 — ELECTRONIC SUPPLEMENTARY MATERIAL [file 13244_2025_2107_MOESM1_ESM.pdf]

Supplementary Material

Supplementary Table 1: MRI Scan Parameters

Parameter	T2WI	T1WI In-phase	T1WI Out-phase	DWI	DCE	HBP
Imaging planes	Axial-coronal	Axial	Axial	Axial	Axial-coronal	Axial-coronal
Fat saturation	fs	-	-	-	SPECIAL	SPECIAL
Sequence	FRFSE	T1WI Dual	T1WI Dual	EPI	LAVA	LAVA
TR(ms)	7500	200	200	10000	3.9	3.9
TE(ms)	85	4.8	2.3	68	1.8	1.8
Flip angle	90	80	80	90	12	12
FOV(mm^2)	400×400	400×400	400×400	400×280	400×400	400×400
Slice thickness	6	6	6	6	6	6
Slice gap	2	2	2	1	-3	-3
Matrix size	288×224	288×160	288×160	128×128	288×224	288×224
b value(s/mm^2)	-	-	-	0,200,800	-	-
Number of acquisition	1	1	1	1	3	1

Supplementary Table 2:FLIS and its three sub-parameters' scoring system

Scoring system	Definition	Score
EnQS: liver-to-kidney signal intensity ratio at HBP 20-min	Hypointense	0
	Isointense	1
	Hyperintense	2
ExQS: Biliary contrast excretion at HBP 20-min	No contrast excretion in intrahepatic or extrahepatic bile ducts	0
	Contrast excretion visible in intrahepatic ducts or left/right hepatic ducts	1
	Contrast excretion visible in common hepatic duct or common bile duct	2
PVQS: Portal vein-to-liver signal intensity ratio at HBP 20-min	Hyperintense	0
	Isointense	1
	Hypointense	2
FLIS	EnQS + ExQS + PVQS	0 ~ 6

Notes: EnQS, enhancement quality score (reflects hepatocyte uptake function); ExQS, excretion quality score (reflects biliary excretion function); PVQS, portal venous quality score (reflects hepatocyte uptake with portal vein clearance); FLIS, functional liver imaging score;HBP, hepatobiliary phase.

Supplementary Table 3：Demographics of study population

Clinical characteristics	Number of cases (n=203)
**Age (range, years)**	49.51±11.79 (23-81)
**Gender**	
Male	133 (65.52%)
Female	70 (34.48%)
**Height (range，cm)**	167.18±7.61 (148-186)
**Weight (range，Kg)**	65.23±10.60 (40-102)
**BMI (range，kg/m^2)**	23.24±2.73 (16.44-31.05)
**Healthy population**	55 (27.09%)
**Liver disease stages**	148 (72.91%)
Chronic liver disease	51 (25.13%)
Compensated cirrhosis	78 (38.42%)
Decompensated cirrhosis	19 (9.36%)
**Etiology of liver disease**	
Hepatitis B	96 (64.87%)
Alcoholic	14 (9.46%)
Non-alcoholic fatty liver disease	12 (8.11%)
Schistosomiasis	1 (0.67%)
Autoimmune	2 (1.35%)
Unknown cause	8 (5.41%)
Mixed	15 (10.14%)
**CTP classification**	97 (47.78%)
A	69 (33.99%)
B	22 (10.84%)
C	6 (2.95%)

Notes: CTP, Child-Turcotte-Pugh; BMI,Body mass index.

Supplementary Table 4: Analysis of differences of clinical data in three groups.

Group	All (n=203)	Control group (n=55)	CLD group (n=51)	Cirrhosis group (n=97)	*P*
Age (years)	49.51±11.79	44.73±13.42	46.47±9.19	53.81±10.50	< 0.001
Gender(n,%)					0.003
Male	133(65.5%)	26(47.3%)	35(68.6%)	72(74.2%)	
Female	70(34.5%)	29(52.7%)	16(31.4%)	25(25.8%)	
BMI(kg/m^2)	23.24±2.73	23.62±2.11	24.01±2.72	22.63±2.92	0.006
TB(μmol/L)	16(12.45,23.7)	13.85(10.08,17.20)	15.45(12.03,19.38)	19.80(14.10,31.10)	< 0.001
ALB(g/L)	41.86±6.24	44.71±2.96	44.67±4.22	38.76±7.02	< 0.001
Cr(μmol/L)	69.31±13.86	67.44±13.74	68.72±14.54	72.45±12.32	0.15
eGFR	102.18±11.83	105.28±11.81	102.31±10.15	100.35±12.39	0.057
INR	1.09±0.22	0.99±0.05	1.00±0.07	1.19±0.27	< 0.001
PT(s)	13.99±2.08	13.05±0.57	13.08±0.81	15.01±2.57	< 0.001
AST(U/L)	28(21,39)	21(17.5,24.5)	24(20.5,32)	36(28,52)	0.006
ALT(U/L)	26(17,38)	17(13,31.5)	28(18,39.5)	30(20,42)	0.051
ALP(U/L)	85(69,114.5)	79(61.5,93)	80(60,92.5)	102(78,139)	< 0.001
GGT(U/L)	33(21,54.5)	24(16,36)	29(19.5,42)	39(26,85)	< 0.001
PLT(10^9/L)	165.30±81.05	236.56±57.62	197.78±55.69	107.81±59.42	< 0.001
EnQS	2(1,2)	2(2,2)	2(2,2)	1(1,2)	< 0.001
ExQS	2(2,2)	2(2,2)	2(2,2)	2(2,2)	NA
PVQS	2(2,2)	2(2,2)	2(2,2)	2(2,2)	NA
FLIS	6(5,6)	6(6,6)	6(6,6)	5(5,6)	< 0.001

Notes: BMI, body mass index; TB, total bilirubin; ALB, albumin; Cr, creatinine; eGFR, estimated glomerular filtration rate; INR, international normalized ratio; PT, prothrombin time; AST, aspartate aminotransferase; ALT, alanine aminotransferase; ALP, alkaline phosphatase; GGT, gamma-glutamyl transferase; PLT, platelet count; EnQS, enhancement quality score; ExQS, excretion quality score; PVQS, portal vein quality score; FLIS, functional liver imaging score; NA, not available.

Supplementary Table 5: Spearman correlation analysis of FLIS and its three sub-parameters with different populations, CTP, and Lab data.

Lab data	r and *P*	EnQS	ExQS	PVQS	FLIS
TB(μmol/L)	r	-0.509	-0.615	-0.586	-0.651
	P	**< 0.001**	**< 0.001**	**< 0.001**	**< 0.001**
ALB(g/L)	r	0.575	0.352	0.616	0.617
	P	**< 0.001**	**< 0.001**	**< 0.001**	**< 0.001**
INR	r	-0.576	-0.558	-0.694	-0.706
	P	**< 0.001**	**< 0.001**	**< 0.001**	**< 0.001**
PT(s)	r	-0.595	-0.566	-0.708	-0.724[†]
	P	**< 0.001**	**< 0.001**	**< 0.001**	**< 0.001**
AST(U/L)	r	-0.437	-0.228	-0.407	-0.459
	P	**< 0.001**	**0.001**	**< 0.001**	**< 0.001**
ALT(U/L)	r	-0.149	0.036	-0.094	-0.159
	P	**0.034**	0.607[*]	0.181[*]	**0.023**
ALP(U/L)	r	-0.469	-0.416	-0.510	-0.545
	P	**< 0.001**	**< 0.001**	**< 0.001**	**< 0.001**
GGT(U/L)	r	-0.404	-0.325	-0.240	-0.391
	P	**< 0.001**	**< 0.001**	**0.001**	**< 0.001**
PLT(10^9/L)	r	0.510	0.168	0.311	0.504
	P	**< 0.001**	**0.016**	**< 0.001**	**< 0.001**
CTP	r	-0.676	-0.617	-0.808	-0.818
	P	**< 0.001**	**< 0.001**	**< 0.001**	**< 0.001**
Population	r	-0.525	-0.198	-0.291	-0.532
	P	**< 0.001**	**0.005**	**< 0.001**	**< 0.001**

Notes: TB, total bilirubin; ALB, albumin; INR, international normalized ratio; PT, prothrombin time; AST, aspartate aminotransferase; ALT, alanine aminotransferase; ALP, alkaline phosphatase; GGT, gamma-glutamyl transferase; PLT, platelet count; EnQS, enhancement quality score; ExQS, excretion quality score; PVQS, portal vein quality score; FLIS, functional liver imaging score.

Symbols: * indicates no correlation; † indicates the strongest correlation.
